# Intracellular drug bioavailability: a new predictor of system dependent drug disposition

**DOI:** 10.1038/srep43047

**Published:** 2017-02-22

**Authors:** André Mateus, Andrea Treyer, Christine Wegler, Maria Karlgren, Pär Matsson, Per Artursson

**Affiliations:** 1 Department of Pharmacy, Uppsala University, BMC, Box 580, Uppsala SE-751 23, Sweden; 2 Cardiovascular and Metabolic Diseases Innovative Medicines, DMPK, AstraZeneca R&D, Mölndal SE-431 83, Sweden; 3 Uppsala University Drug Optimization and Pharmaceutical Profiling Platform (UDOPP), Department of Pharmacy, Uppsala University, Box 580, Uppsala SE-751 23, Sweden; 4 Science for Life Laboratory Drug Discovery and Development platform (SciLifelab DDD-P), Uppsala University, Uppsala SE-751 23, Sweden

## Abstract

Intracellular drug exposure is influenced by cell- and tissue-dependent expression of drug-transporting proteins and metabolizing enzymes. Here, we introduce the concept of intracellular bioavailability (F_ic_) as the fraction of extracellular drug available to bind intracellular targets, and we assess how F_ic_ is affected by cellular drug disposition processes. We first investigated the impact of two essential drug transporters separately, one influx transporter (OATP1B1; *SLCO1B1*) and one efflux transporter (P-gp; *ABCB1*), in cells overexpressing these proteins. We showed that OATP1B1 increased F_ic_ of its substrates, while P-gp decreased F_ic_. We then investigated the impact of the concerted action of multiple transporters and metabolizing enzymes in freshly-isolated human hepatocytes in culture configurations with different levels of expression and activity of these proteins. We observed that F_ic_ was up to 35-fold lower in the configuration with high expression of drug-eliminating transporters and enzymes. We conclude that F_ic_ provides a measurement of the net impact of all cellular drug disposition processes on intracellular bioavailable drug levels. Importantly, no prior knowledge of the involved drug distribution pathways is required, allowing for high-throughput determination of drug access to intracellular targets in highly defined cell systems (e.g., single-transporter transfectants) or in complex ones (including primary human cells).

Drug-transporting proteins and metabolizing enzymes are determinants of intracellular drug disposition^1^,^2^: uptake transporters increase the drug concentration in the cell interior^3^,^4^,^5^, while efflux transporters^6^,^7^,^8^ and enzymes^9^,^10^ reduce the amount of intracellularly available compound (Fig. 1). Therefore, these proteins are of major importance for drug efficacy and toxicity^11^. The contribution of such drug-disposition proteins has been studied *in vitro*, e.g., in cell lines expressing specific transporters^5^,^12^,^13^,^14^,^15^, in hepatocytes (in suspension, monolayer and sandwich cultures)^16^,^17^, and in more complex systems, such as tissue slices^18^ and 3D cultures^19^. Typically, only the relative impact of transporters on the intracellular compound concentration is reported, i.e., fold-differences in intracellular concentration when the transporter or metabolic enzymes are active (or overexpressed) compared to when these proteins are inhibited (or absent). Quantitative determination of the intracellular unbound drug concentration, which is the relevant concentration for binding to intracellular target proteins, either relies on experimentally demanding setups in freshly isolated cells^3^,^4^,^5^,^20^,^21^ or tissues^22^,^23^, or on *a priori* knowledge of transport and metabolic mechanisms for the compound being studied^24^. To overcome these limitations, we recently developed a simple methodology to measure intracellular unbound drug concentrations in cultured cells^25^, compatible with high-throughput formats^26^,^27^. We also showed that our methodology correlates with binding to an intracellular target (thymidylate synthase)^28^.

In this work, we apply this methodology to study the impact of drug-transporting proteins and metabolizing enzymes on intracellular unbound compound concentrations. For this purpose, we introduced the concept of intracellular compound bioavailability (F_ic_), which is the fraction of the externally added compound concentration that is available to bind targets in the cell interior. Throughout this study, this concept is equivalent to the unbound drug accumulation ratio (Kp_uu_), a term commonly used in pharmacokinetic studies of blood-to-tissue concentration ratios^22^. However, there are situations in which the Kp_uu_ term is not applicable (e.g., when experiments are performed in the presence of serum proteins). First, we evaluated how F_ic_ is affected by a single uptake or a single efflux transporter. We used well defined cell lines overexpressing the organic anion-transporting polypeptide 1B1 (OATP1B1; SLCO1B1*1a) and P-glycoprotein (P-gp; *ABCB1*), two of the transporters most commonly involved in drug disposition^1^. We then studied the parallel impact of multiple transporters and enzymes in a more complex system, freshly isolated human hepatocytes. The hepatocytes were used in two configurations known to display different gene expression and activity of transporters and enzymes: directly after isolation and after 24 h of culture in monolayer format^29^,^30^,^31^. In conclusion, our simple methodology provides a good estimate of compound available to bind targets in the cell interior, even in complex cell systems.

## Results

### Impact of OATP1B1 on intracellular bioavailability (F_ic_) of compounds

**Figure 1:**
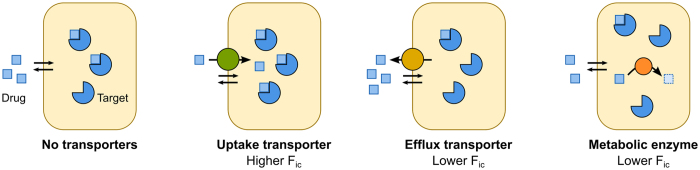
Impact of drug-transporting proteins and metabolizing enzymes on intracellular drug bioavailability (F_ic_, the fraction of externally added drug that is available to bind targets in the cell interior). Uptake transporters (green, e.g. OATP1B1) increase the F_ic_, while efflux transporters (light orange, e.g. P-gp) and metabolizing enzymes (dark orange, e.g. CYP3A4) lower the F_ic_. Arrows represent diffusion over the plasma membrane (passive lipoidal transmembrane diffusion or carrier-mediated diffusion).

**Figure 2:**
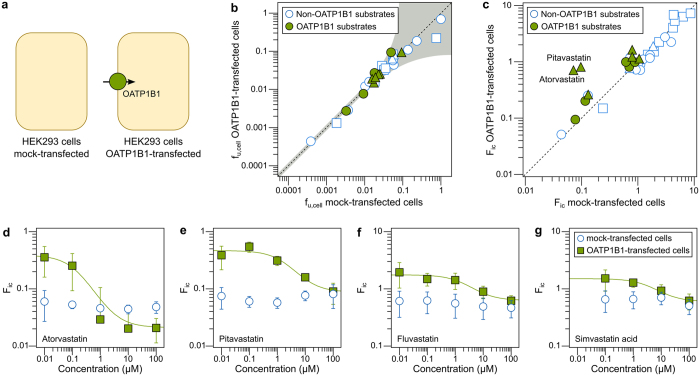
Impact of the uptake transporter OATP1B1 on intracellular bioavailability (F_ic_). (**a**) Schematic representation of cell types used in this study: mock-transfected HEK293 cells express negligible levels of relevant drug-transporting proteins; OATP1B1-transfected HEK293 cells express OATP1B1. (**b**) Comparison between f_u,cell_ in mock-transfected and OATP1B1-transfected HEK293 cells. Shaded area represents the impact of a theoretical error on f_u,cell_ from measurements of f_u,hom_ with 15% error. (**c**) Comparison between F_ic_ in mock-transfected and OATP1B1-transfected HEK293 cells at 0.1 μM compound concentration. In **b** and **c**, negatively charged compounds at pH 7.4 are represented as triangles, neutral and zwitterionic species are represented by circles, and positively charged compounds are represented by squares. Substrates of OATP1B1 are highlighted in green. (**d**–**g**) Concentration-dependence of F_ic_ in OATP1B1-transfected HEK293 cells (green filled squares), fitted with a sigmoidal model (green line), and mock-transfected HEK293 cells (blue circles) for atorvastatin (**d**), pitavastatin (**e**), fluvastatin (**f**), and simvastatin acid (**g**). For simvastatin acid, intracellular concentrations at 0.01 μM were below limit of quantification.

We investigated the impact of drug-transporting proteins on intracellular compound bioavailability (F_ic_). First, we studied the influence of an important uptake transporter (Fig. 1) by using HEK293 cells transfected with human OATP1B1 or with an empty vector (mock-transfected cells; Fig. 2a). We measured the intracellular fraction of unbound compound (f_u,cell_) in cell homogenates and cellular compound accumulation (Kp) in live cells (see ‘Methods’ for details). From these parallel experiments, F_ic_ was then calculated as the product of f_u,cell_ and Kp, providing a measure of how much of an extracellularly applied drug concentration is freely available in the cell interior.

Measurements of f_u,cell_ showed no significant difference between mock- and OATP1B1-transfected cells (Fig. 2b; Supplementary Table 2; r_S_ = 0.97). This indicates a negligible contribution from OATP1B1 to the total binding to cellular proteins, which can be explained by previous observations that a non-saturable component, most likely the membrane lipids, is the major cellular binding site^25^,^32^,^33^,^34^. Further, OATP1B1 contributes only 0.03% of all protein in the transfected cells (12 pmol/mg membrane protein^35^, assuming membrane proteins to be 30% of the total cellular protein mass^36^).

In contrast, substrates of OATP1B1 showed higher total cellular accumulation (Kp) in OATP1B1- than in mock-transfected cells (Supplementary Table 2). This difference was also reflected in F_ic_, which was, on average, 2.9-fold higher in OATP1B1-transfected cells (range: 1.1–9.8-fold; *p* = 0.001 in Wilcoxon matched-pairs signed rank test) (Fig. 2c). In contrast, non-substrates of OATP1B1 showed similar F_ic_ in both cell lines (range: 0.57–1.9-fold difference between OATP1B1- and mock-transfected cells; *p* = 0.06 in Wilcoxon matched-pairs signed rank test). Atorvastatin and pitavastatin displayed the largest increase in F_ic_ from mock-transfected cells (9.8 and 8.3-fold higher in OATP1B1-transfected cells, respectively). This is in agreement with that OATP transport is the rate-limiting step in the liver disposition of these statins^12^,^13^,^37^,^38^. The impact of the uptake transporter decreased with increasing lipophilicity of the compounds (r_S_ = −0.63, p = 0.04 for the rank-order correlation between difference in F_ic_ and log D of substrates; Supplementary Figure 1b). This most likely reflects the well-established increase in transmembrane diffusion with increasing lipophilicity^39^,^40^,^41^.

To further investigate if the observed differences were caused by OATP1B1-mediated uptake, we measured F_ic_ for four substrates (atorvastatin, pitavastatin, fluvastatin, and simvastatin acid) across a range of concentrations (Fig. 2d–g). In mock-transfected HEK293 cells, which have negligible basal expression of drug-transporting proteins^42^, F_ic_ was independent of compound concentration for all substrates, indicating that passive permeability across the cell membrane was the main uptake mechanism in these cells. In contrast, in OATP1B1-transfected cells, F_ic_ decreased with increasing compound concentration, approaching levels in mock-transfected cells at higher concentrations. The concentrations at which F_ic_ was half of its maximal value were close to previously reported K_m_ values for OATP1B1-mediated transport of the respective substrates^12^,^13^,^43^, reflecting saturation of the transporter and an ensuing dominance of a passive mechanism.

### Impact of P-gp on intracellular bioavailability (F_ic_) of compounds

**Figure 3:**
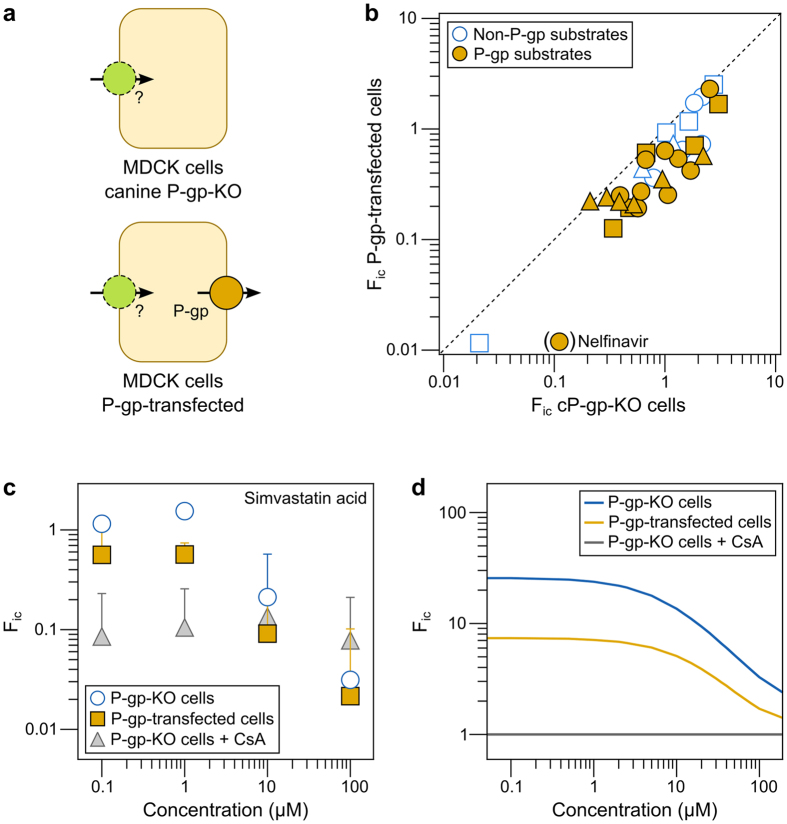
Impact of the efflux transporter P-gp on intracellular bioavailability (F_ic_). (**a**) Schematic representation of cell types used in this study: MDCK cells where canine P-gp has been knocked-out using CRISPR/Cas 9 (cP-gp-KO); MDCK cells transfected with human P-gp, expressing both canine and human P-gp. (**b**) Comparison between F_ic_ in cP-gp-KO and in P-gp-transfected MDCK cells at 0.5 μM compound concentration. Negatively charged compounds at pH 7.4 are represented as triangles, neutral and zwitterionic species are represented by circles, and positively charged compounds are represented by squares. Substrates of P-gp are highlighted in yellow. Nelfinavir is represented in parentheses as its F_ic_ is outside the range of this plot in P-gp-transfected cells. (**c**) Relationship between simvastatin acid concentration and F_ic_ in P-gp-transfected MDCK cells (yellow filled squares), P-gp-KO MDCK cells (blue circles), and P-gp-KO MDCK cells pre-incubated (15 min) followed by co-incubation with 10 μM cyclosporine A (gray triangles). (**d**) Kinetic cell model simulations that best described experimental observations of concentration-dependence F_ic_ for simvastatin acid (conditions: passive permeability = 10 × 10^−6^ cm/s; V_max,uptake_ = 1000 pmol/min/mg protein; K_m,uptake_ = 10 μM; V_max,efflux_ = 1000 pmol/min/mg protein; K_m,efflux_ = 100 μM).

We next evaluated how efflux transporters affect F_ic_ (Fig. 1) through measurements in MDCK cells transfected with human P-gp. To determine baseline F_ic_ in these cells in the absence of P-gp, we used wild-type MDCK cells where background endogenous canine P-gp was knocked out using CRISPR-Cas9 technology (cP-gp-KO)^44^ (Fig. 3a)—see ‘Supplementary results’ for a discussion on the choice of these cells as a control.

As in the experiments with HEK293 cells, we first measured f_u,cell_ in MDCK cells transfected with human P-gp (Supplementary Table 3). We used this value of f_u,cell_ for all MDCK cell types used in this study, based on our observation above that the low levels at which transporters are expressed (compared to total protein) do not affect binding (Fig. 2b; P-gp contributes only 0.1% of all protein in P-gp-transfected MDCK cells (6.0 pmol/mg total protein, in-house data)).

We then combined f_u,cell_ and Kp measurements to calculate F_ic_ in P-gp-transfected MDCK cells and in cP-gp-KO cells (Supplementary Table 3). Because of the active efflux of substrates when the transporter is present, the F_ic_ of compounds previously reported to be P-gp substrates was on average 2.0-fold lower in P-gp-expressing than in cP-gp-KO cells (range: 0.94–20-fold lower in P-gp transfected than cP-gp-KO cells; *p* < 0.001 in Wilcoxon matched-pairs signed rank test; Fig. 3b). For compounds not previously described as substrates, F_ic_ was more similar in the two cell lines (range: 1.1–3.0-fold difference between cP-gp-KO cells and P-gp transfected cells), but significantly different (*p* = 0.001; see discussion below). Nelfinavir, an established P-gp substrate, displayed a remarkably low F_ic_ in P-gp-transfected cells (20-fold lower than in cells not expressing P-gp). This indicates that P-gp contributes strongly to the cellular elimination of nelfinavir, in particular in cells that do not metabolize this compound. Overall, lipophilic molecules showed lower F_ic_ in P-gp transfected cells (r_S_ = −0.36, p = 0.05 for the rank-order correlation between difference in F_ic_ and log D of all compounds; Supplementary Figure 2b), independently of being previously described as substrates or not. This possibly reflects that compounds bind P-gp from within the inner leaflet of the plasma membrane^45^,^46^, and suggests that additional compounds in our dataset may be substrates of P-gp, besides those previously reported as such.

As for the OATP1B1 substrates, we measured the concentration-dependence of F_ic_ in P-gp-expressing and cP-gp-KO cells. For simvastatin acid, F_ic_ in P-gp-expressing cells was lower than that in cP-gp-KO cells at low concentrations and approached it at higher concentrations (Fig. 3c). Surprisingly, F_ic_ decreased with increasing compound concentration in both the P-gp expressing cells and in the cP-gp-KO cells, which was not caused by a saturation of binding sites^25^ (Supplementary Figure 4). Similar results were observed for loperamide (Supplementary Figure 3). When inhibiting active transport with 10 μM cyclosporine A (a general transporter inhibitor^47^), we observed that F_ic_ of simvastatin acid was constant across all concentrations and lower than previously observed without inhibition (Fig. 3c). This suggests that additional active transport mechanisms (including uptake transporters) are present in these cells and affect the cellular disposition of this compound.

To better understand these observations, we simulated cellular drug accumulation using three simple kinetic models: 1) one including both active uptake and active efflux mechanisms (corresponding to P-gp transfected cells), 2) one including only the uptake mechanisms (corresponding to cP-gp-KO cells), and 3) one including only the passive mechanism (corresponding to inhibition of all active mechanisms with cyclosporine A). We simulated all possible combinations of low, medium and high values of passive permeability (0.1, 1, and 10 × 10^−6^ cm/s), V_max_ (10, 100, and 1000 pmol/min/mg protein) and K_m_ (1, 10, and 100 μM) for each of the transporters (see Matsson *et al*.^48^ and the ‘Methods’ section for details on the simulations). The simulations were in best agreement with the experimental observations for a compound with high passive permeability (10 × 10^−6^ cm/s), medium affinity (K_m_ = 10 μM) for an uptake transporter and lower affinity (K_m_ = 100 μM) for an efflux transporter, with both transporters having comparable (high) transport capacities (V_max_) (Fig. 3d). This matches previous observations for simvastatin acid, which show that this compound, in addition to being an OATP1B1 substrate, has a high passive permeability, and a low affinity for efflux transporters^49^,^50^,^51^.

### Intracellular bioavailability (F_ic_) in complex cell systems

**Figure 4:**
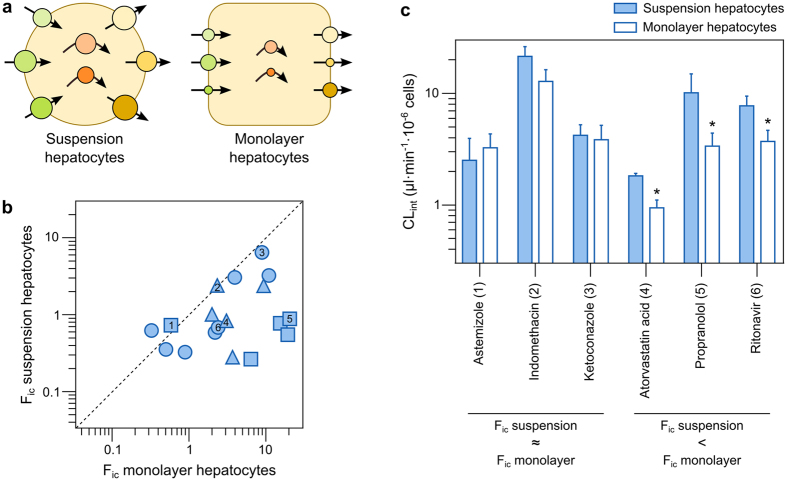
Impact of multiple transporters and metabolizing enzymes on intracellular bioavailability (F_ic_). (**a**) Schematic representation of hepatocyte cultures used in this study: freshly isolated human hepatocytes in suspension show higher activity of drug-transporting proteins and metabolizing enzymes than hepatocytes cultured for 24 h in monolayer format. (**b**) Comparison between F_ic_ in suspension hepatocytes and hepatocytes cultured in monolayer format. Negatively charged compounds at pH 7.4 are represented as triangles, neutral and zwitterionic species are represented by circles, and positively charged compounds are represented by squares. Compound numbers indicate: 1) astemizole; 2) indomethacin; 3) ketoconazole; 4) atorvastatin acid; 5) propranolol; 6) ritonavir. (**c**) Differences in metabolic clearance in suspension hepatocytes and hepatocytes cultured in monolayer format. *p < 0.05 in Mann-Whitney test comparing suspension and monolayer hepatocytes.

Our results in MDCK cells revealed that unknown endogenous transport mechanisms can influence F_ic_ to a larger extent than previously recognized. To further investigate how multiple transporters impact F_ic_, we performed measurements in freshly isolated human hepatocytes. These cells also express metabolizing enzymes, which can further affect F_ic_ (Fig. 1), and better represent an *in vivo* situation where multiple processes occur simultaneously. Hepatocytes were used in two commonly employed culture conditions (Fig. 4a): 1) freshly isolated cells (in suspension) that express similar levels of transporters and metabolizing enzymes as those observed in the human liver^29^,^30^,^31^,^52^; and 2) cells cultured for 24 h post-isolation (in monolayer format) with a significant down-regulation of many important transporters and enzymes due to cell dedifferentiation^29^,^30^,^31^. Thus, without knowing the exact composition of the contributing transporters and enzymes, their relative impact in two complex hepatocyte systems could be compared. For these studies, we selected a subset of 16 compounds from the initial dataset (Supplementary Table 4). The compounds were chosen to be structurally diverse and to be substrates of different transporters and enzymes with altered expression (Supplementary Table 5).

On average, F_ic_ was 5.7-fold lower in freshly isolated suspension hepatocytes than in monolayer hepatocytes cultivated for 24 h (Fig. 4b; *p* = 0.0002 in Wilcoxon matched-pairs signed rank test). No apparent correlation was observed between the physicochemical characteristics of the compounds and the differences in F_ic_ in the two types of hepatocytes (Supplementary Figure 5). However, compounds that are reported as substrates of multiple efflux transporters and enzymes displayed larger differences between the two culturing conditions (r_S_ = 0.72 for the rank-order correlation between differences in F_ic_ and the number of targeted enzymes and transporters). This was in agreement with the reported higher mRNA transcript levels in suspension hepatocytes for these proteins^29^,^30^,^31^, which are associated with the removal of compounds from the cell interior (leading to a lower F_ic_). In agreement with these results, compounds that were metabolically cleared at higher rates (e.g., atorvastatin, propranolol, and ritonavir; Fig. 4c; *p* < 0.05 in Mann-Whitney test) showed lower F_ic_ in suspension hepatocytes. In analogy, compounds with a similar metabolic clearance under both culture conditions (e.g., astemizole, indomethacin, and ketoconazole) also had similar F_ic_ (Fig. 4c). These results strongly support our hypothesis that F_ic_ is a general predictor of intracellular drug disposition. We also measured the protein concentrations of the major transporters and metabolic enzymes involved in the clearance of the studied compounds using state-of-the-art mass spectrometry-based proteomics (Supplementary Figure 6). To our surprise, the agreement between mRNA transcript levels^29^,^30^, enzymatic activity (Fig. 4c) and F_ic_ (Fig. 4b) did not translate to the protein level, since we did not observe significant differences in protein concentrations between the two culturing conditions (*p* = 0.6 in Wilcoxon matched-pairs signed rank test). This can be explained by drug-transporting proteins being redistributed from the plasma membrane to intracellular compartments under certain culturing conditions, or as a result of external stimuli^53^,^54^,^55^. Similarly, the activity of metabolic enzymes can be reduced by post-translational modifications^56^ or lower levels of co-factors^57^. In these cases, these proteins will contribute to the whole-cell protein levels but will not affect F_ic_. This underscores the importance of functional studies to assess the impact of drug-transporting proteins and metabolizing enzymes on intracellular drug concentrations.

## Discussion

Intracellular compound exposure is of paramount importance for the pharmacodynamics of drugs acting on intracellular targets, and for interactions with intracellular drug-metabolizing enzymes^11^,^28^. In this study, we introduce the concept of intracellular compound bioavailability (F_ic_) and demonstrate its utility in describing the impact of drug-transporting proteins and metabolizing enzymes on cellular drug disposition.

Our methodology is based on the parallel determination of the fraction of intracellular compound that is not bound to cellular components (f_u,cell_) and cellular compound accumulation (Kp). Our results indicate that overexpression of single proteins (e.g. OATP1B1) has little impact on f_u,cell_ (Fig. 2b). Further, good correlations were observed for f_u,cell_ across all cell types used in this study, despite different proteomes in these cells (r_S_ > 0.8 for all comparisons of between HEK293 cells, MDCK cells and freshly isolated hepatocytes). This supports previous observations that f_u,cell_ is mostly dominated by membrane partitioning^25^,^26^,^32^,^33^,^34^, and that specific proteins contribute negligibly to this parameter.

In contrast, clear effects were observed for F_ic_ when HEK293 cells were transfected with the cellular uptake-mediating transporter OATP1B1, with intracellular exposure increasing for all investigated substrates of OATP1B1. Interestingly, some statins, such as atorvastatin and pitavastatin, showed F_ic_ < 0.1 when the transporter was lacking, suggesting that they have limited cell penetration in the absence of transporters.

Our studies on the concentration-dependent uptake of the OATP1B1 substrates showed a greater impact of the transporter at lower substrate concentrations. At the lowest measured concentrations (approaching unbound clinical plasma concentrations), F_ic_ was up to 10-fold higher for atorvastatin and pitavastatin, and up to 2-fold higher for fluvastatin and simvastatin acid in the presence of the transporter. These results are in agreement with clinical drug-drug interaction studies^12^,^13^,^37^,^38^, in which the pharmacokinetics of atorvastatin and pitavastatin are more affected than fluvastatin and simvastatin acid when co-administered with OATP1B1 inhibitors. For example, cyclosporine A increases the AUC of pitavastatin and atorvastatin by 5- to 15- fold, and that of simvastatin and fluvastatin by 3- to 4-fold in human subjects^58^. Furthermore, pharmacogenomic studies have also shown that reduced-function haplotypes of *SLCO1B1* contribute to higher plasma concentrations of these compounds^59^,^60^,^61^. With the exception of simvastatin acid, which (based on only one study^62^) has a larger clinical effect than the other statins from a reduced function polymorphism of OATP1B1, our results accurately reproduce the rank order of the clinically observed AUC change (pitavastatin > atorvastatin > fluvastatin).

Contrary to HEK293 cells, MDCK cells express significant levels of endogenous drug-transporting proteins, including canine P-gp, that complicate the interpretation of *in vitro* transport studies^42^,^63^. To abolish the impact of canine P-gp in the present study, we used our recently established MDCK cell line where this transporter was completely knocked out using CRISPR-Cas9 (cP-gp-KO cells)^44^. This enabled the study of baseline transport into MDCK cells without the need for chemical inhibition of P-gp. This was considered important, since we have shown that P-gp inhibitors such as elacridar also inhibit other endogenous transporters^35^,^47^. Since efflux transporters are driven by intracellular (or intramembraneous) substrate concentrations, it is desirable to avoid simultaneous inhibition of the uptake transporters that some compounds depend on to reach the cell interior. Thus, studies that rely on chemical inhibition can misclassify compounds as non-substrates. In fact, we observed that some compounds not previously annotated as P-gp substrates (e.g. astemizole, ketoconazole, lovastatin and rosiglitazone) showed large differences in F_ic_ between P-gp-expressing and cP-gp-KO cell lines. These compounds have previously been reported as P-gp inhibitors^64^,^65^,^66^. Our findings suggest that they are also transported substrates. In follow-up experiments, we have confirmed that at least two of these compounds show asymmetrical transport across a monolayer of MDCK cells (manuscript in preparation). This indicates that the setup used here can replace more demanding transport assays to identify substrates of proteins involved in drug disposition and resistance to chemotherapy^67^.

The possibility of studying the involvement of multiple transport processes with this methodology, as observed with simvastatin acid in MDCK cells (Fig. 3c), prompted us to study F_ic_ in the more complex system of freshly isolated human hepatocytes. Primary human hepatocyte models are extensively used in drug discovery and are available in many different configurations, which display marked differences in gene expression and activity of drug-transporting proteins and drug-metabolizing enzymes^29^,^30^,^31^. Our measurements of F_ic_ reflected these differences in that lower F_ic_ values were observed in suspension hepatocytes, where the elimination pathways via efflux transporters and drug-metabolizing enzymes are more active^29^,^30^,^31^. Interestingly, only compounds reported to be substrates of multiple drug-transporting proteins and metabolizing enzymes were affected (Supplementary Table 5), while compounds reported to be substrates of mainly one or two metabolizing enzymes (e.g. astemizole, indinavir, ketoconazole, lopinavir, and rosiglitazone) displayed similar F_ic_ in both culturing conditions. For example, ritonavir, a compound with an F_ic_ of 1.0 in mock-transfected HEK293 cells (where passive mechanisms are the major determinants of drug accumulation^25^), showed an F_ic_ of 0.26 in freshly isolated suspension hepatocytes (indicative of a dominance of elimination mechanisms) and an F_ic_ of 2.3 in monolayer hepatocytes (indicative of reduced elimination and a dominance of active uptake mechanisms). This was confirmed by functional *in vitro* clearance studies, which showed that the metabolic CL_int_ of ritonavir was in fact reduced in monolayer hepatocytes (Fig. 4c). Thus, our results indicate that F_ic_ can be used to assess intracellular drug exposure also in complex systems with multiple sequential and parallel drug disposition processes.

Previous approaches to determine intracellular unbound drug concentrations based on mathematical modeling require extensive collection of experimental data (at multiple time points and concentrations)^3^,^4^,^21^, which limits their compound throughput. Alternative approaches rely on a separate measurement of drug binding to the cells that is combined measurements of the total intracellular drug concentrations. In such approaches, binding can be estimated by inhibiting all active processes (with chemical inhibitors^24^,^68^ or reduced temperature^21^) or using equilibrium dialysis of tissue or cell homogenates^22^,^25^. The use of chemical inhibitors assumes that all transport and metabolism mechanisms are known and can be inhibited, while lowering the temperature assumes that changes in membrane fluidity^69^ do not affect drug binding. Our approach uses equilibrium dialysis of cell homogenates and assumes that binding is not affected by the disruption of the cellular context during homogenization. This allows the processing of large numbers of compounds without prior knowledge of compound elimination kinetics or metabolic pathways. Despite the utility of a simple estimate of intracellular available compound, the F_ic_ parameter does not provide information on the subcellular distribution of the compound. Instead, it provides an average bioavailability in the whole cell. As previously shown, mathematical modeling of pH^22^,^25^ or electrochemical gradients^5^ can offer insight into the accumulation of compounds in lysosomes or mitochondria. In addition, inhibition of ion channels responsible for the maintenance of these gradients can be used to validate such models^25^. Alternatively, subcellular fractionation can be performed prior to^70^ or after^20^ incubation with the drug. However, fractionation approaches might alter compound distribution, since the organelle is no longer in its native environment. All of these approaches are compatible with the overall methodology presented in this study and can be used in specific cases, when the target of interest is located in a particular subcellular compartment.

In conclusion, we introduce F_ic_ as a new approach to study the impact of proteins involved in drug disposition on the intracellular drug concentration available for target and off-target (e.g. drug-metabolizing enzymes) interactions. We show how single and multiple drug-transporting proteins and metabolizing enzymes influence F_ic_ in various cellular systems of different complexity. We also show that our setup enables identification of potential substrates of transporters and could constitute an alternative to state-of-the-art *in vitro* permeability assays. Thus, F_ic_ provides a true value of intracellular accumulation that is unbiased from membrane interactions. F_ic_ should therefore not only be applicable in predictive pharmacokinetics, but in particular during compound profiling in drug discovery aiming at intracellular targets^11^,^27^,^28^,^71^,^72^.

## Methods

### Theoretical background: intracellular compound bioavailability (F_ic_)

To determine intracellular unbound compound concentrations, we measured intracellular fraction of unbound compound (f_u,cell_) in parallel with total intracellular compound accumulation (C_cell_), as described below. Intracellular compound bioavailability (F_ic_) was calculated as the ratio between intracellular unbound compound concentration (C_u,cell_ = f_u,cell_∙C_cell_) (the concentration available to bind intracellular targets) and the externally added compound concentration (C_medium_):


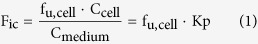


where Kp is the ratio C_cell_/C_medium_.

In our previous work^25^, this ratio was termed unbound drug accumulation ratio (Kp_uu_), in analogy to previous work on unbound drug exposure in brain tissue^73^. We introduce the more descriptive and general F_ic_ parameter here, since it is applicable also to situations where only a fraction of the extracellular compound is unbound. This is of interest in drug discovery settings where cellular screens are often performed in the presence of serum proteins. In such assay setups, the Kp_uu_ term is not applicable, since it assumes extracellular drug to be completely unbound.

Contrary to oral bioavailability, which is defined in terms of dose (i.e., amount), F_ic_ is defined in concentration terms. For that reason, F_ic_ can take any positive value and is not limited to the range between zero and one. However, F_ic_ can be interpreted in similar terms, as the fraction of the extracellular concentration that is available intracellularly.

### Materials and compound set

Cell culture reagents were acquired from Thermo Fisher Scientific Inc. or Sigma-Aldrich. HEK293 cells were originally obtained from Thermo Fisher Scientific and MDCK cells from ATCC. Test compounds of analytical grade (≥95% purity) were obtained from Sigma-Aldrich, Toronto Research Chemicals or OChem Inc. Compounds were dissolved in dimethyl sulfoxide (DMSO) and stored at −20 °C.

The compound set used in this study included 34 drug-like molecules (Supplementary Table 1), and was enriched in compounds previously described as OATP1B1 (n = 11)^74^ or P-gp substrates (n = 22)^75^,^76^, with ten of the compounds included being substrates of both drug-transporting proteins. ADMET Predictor, version 7.0 (SimulationsPlus, Lancaster, CA) was used to predict physicochemical characteristics of the compound set.

The compounds were generally within the boundaries of Lipinski’s rule of 5 (64% of molecules within all rule-of-5 boundaries^77^), with the major violation being MW > 500 Da (83% of violations). At physiological pH (pH = 7.4), eight of the included compounds were predicted to be predominantly negatively charged, fourteen as mostly uncharged, nine as mostly positively charged, and three as zwitterions (Supplementary Table 1). In agreement with the literature^74^, substrates of OATP1B1 were generally negatively charged and more polar than non-substrates (mean PSA of 105 vs. 74 Å^2^; *p* = 0.03). Compounds previously described as substrates of P-gp were more polar (mean PSA of 99 vs. 56 Å^2^; *p* = 0.002) and larger (mean MW of 497 vs. 335; *p* < 0.001) than compounds lacking reports of P-gp-mediated transport.

### Cell culture and preparation of experiments

#### Cell line maintenance

HEK293 cells stably transfected with OATP1B1 (SLCO1B1*1a allele) or with the corresponding empty vector were maintained in Dulbecco’s modified Eagle’s medium (DMEM) supplemented with 10% fetal bovine serum (FBS), 2 mM L-glutamine and 75 μg/ml hygromycin B.^78^ CRISPR-Cas9 canine P-gp knock-out MDCK cells (cP-gp-KO) were generated as described elsewhere^44^. MDCK cells were grown in DMEM (glucose 1 g/l) supplemented with 10% FBS, GlutaMAX, penicillin (100 units/ml), and streptomycin (100 μg/ml). For human P-gp (*ABCB1*) transfected MDCK cells, hygromycin B (375 μg/ml) was included as a selection antibiotic. All cell lines were kept at 37 °C in a humidified 5% CO_2_ atmosphere and subcultivated twice a week with a 1:6 ratio.

#### Isolation of primary human hepatocytes

Hepatocytes were isolated from human liver tissue obtained from two donors undergoing surgical resections at the Department of Surgery, Uppsala University Hospital. Ethical approval was granted by the Uppsala Regional Ethics Committee (ethical approvals no. 2009/028 and 2011/037), donors gave informed consent and all studies were performed in accordance with the current national regulations and ethical guidelines. Isolation was performed using a two-step collagenase procedure, as previously described^52^,^79^.

#### Preparation of cell homogenates for cell binding experiments

For intracellular fraction of unbound compound experiments, cell lines or primary cells were suspended to a concentration of 10 × 10^6^ cells/ml in Hank’s balanced salt solution (HBSS). Cell suspensions were homogenized for 10 s with a VCX-500 ultrasonic processor (Sonics & Materials) at 20% intensity and used immediately for binding experiments^25^.

#### Preparation for intracellular compound accumulation experiments

HEK293 cells were grown for two days in 24-well plates, as previously described^25^. MDCK cells were grown for two days in 96-well plates, prior to the intracellular compound accumulation experiments. The growth media were the same as described in the section ‘Cell line maintenance’ without addition of hygromycin B to avoid transporter inhibition.

For the corresponding experiments in human hepatocytes, two cell culture configurations were used: freshly isolated cells in suspension, or cells cultivated as monolayers for 24 h. The freshly isolated cells were suspended directly after isolation at a concentration of 5 × 10^6^ cells/ml in HBSS. The cell suspension (100 μl) was added to 96-well plates, which were immediately used for accumulation experiments. The monolayer-cultured hepatocytes were allowed to attach to collagen-coated 24-well plates for three hours (375 000 cells/well) in DMEM with HEPES supplemented with 5% FBS, penicillin (100 units/ml), streptomycin (100 μg/ml), insulin (4 μg/ml), and dexamethasone (1 μM). After cell attachment, medium was replaced by hepatocyte maintenance medium (Lonza) supplemented with insulin (10 μg/ml), transferrin (5.5 μg/ml), selenium (5 ng/ml), penicillin (100 units/ml), streptomycin (100 μg/ml), and dexamethasone (0.1 μM). Cells were kept in maintenance medium for 24 h, until the start of the intracellular compound accumulation experiments.

### Intracellular fraction of unbound compound

Binding to cell homogenates at steady-state was measured using dialysis as previously described^26^. Simultaneous measurement of intracellular binding for multiple compounds (cassette-mode) is made possible by our previous observation that binding to cellular structures at low compound concentration is mainly non-specific^25^ and unaffected by the presence of other drug-like molecules^26^. Briefly, the cell homogenate was spiked with six randomly-chosen compounds. Homogenates were dialyzed for 4 h using a Rapid Equilibrium Dialysis device (Thermo Fisher Scientific Inc.). At the end of the incubation, uniform sample matrices were obtained by addition of blank homogenate to samples from the buffer chamber, and by addition blank buffer to samples from the homogenate chamber. Dilutions of samples from the homogenate chambers (10-fold and 100-fold) were prepared. Protein was precipitated with acetonitrile/water (60:40) spiked with 50 nM warfarin (internal standard). Samples were centrifuged for 20 min at 2465 × *g*. Compounds in the supernatant were analyzed by ultra-performance liquid chromatography coupled to tandem mass spectrometry (UPLC-MS/MS), as described below.

The fraction of compound that was not bound to cell homogenate (f_u,hom_) was calculated according to:


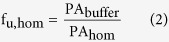


where PA_buffer_ is the peak area of compound in the buffer chamber and PA_hom_ is the peak area of compound in the homogenate chamber, both corrected for the peak area of the internal standard in the respective chamber. For each compound, the dilution of the homogenate sample where PA_hom_ was closest to PA_buffer_ was used.

The fraction of unbound compound in the cell (f_u,cell_) was calculated according to:


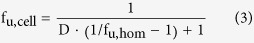


where D was estimated for each homogenate preparation based on a cellular volume of 6.5 μl/mg protein^80^, and on the protein concentration measured using the BCA Protein Assay Reagent Kit (Thermo Fisher Scientific Inc.).

### Intracellular compound accumulation

Intracellular compound accumulation at steady-state was measured essentially as described before^25^. Briefly, cells were washed twice with HBSS and incubated for 45 min with solutions of single test compounds (in HBSS) on an orbital shaker (300 rpm) at 37 °C. At the end of the experiment, the remaining extracellular compound solutions were collected and cells were washed twice with ice-cold phosphate-buffered saline pH 7.4 (PBS). For hepatocytes in suspension, the cells were separated from the extracellular solutions by centrifugation (100 × *g* for 5 min). Compounds were extracted from the cells using acetonitrile/water (60:40) spiked with 50 nM warfarin (internal standard). Extracellular medium samples were diluted 10-fold with the same extraction solution. All samples were centrifuged for 20 min at 2465 × *g* and compounds were quantified in the supernatant by UPLC-MS/MS, as described below. Protein concentrations were measured in representative wells of the plates using the BCA Protein Assay Reagent Kit (Thermo Fisher Scientific Inc.).

The ratio between total compound concentrations in the cells and in the medium (Kp) was calculated according to:


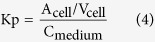


where A_cell_ is the amount of compound in the cell samples, V_cell_ is the cell volume (6.5 μL/mg protein^80^), and C_medium_ is the compound concentration in the medium sample.

### Metabolic clearance (CL_int_) in primary human hepatocytes

Metabolic clearance (CL_int_) was determined, as previously described^81^. Briefly, freshly isolated human hepatocytes (in suspension or after 24 h of culture in monolayer format) were incubated with 1 μM compound solution (total incubation volume = 200 μl). At time points 0, 10, 30, and 60 min, the reaction was stopped by addition of acetonitrile (200 μl) spiked with 50 nM warfarin (internal standard). Samples were then centrifuged for 20 min at 2465 × *g* and compounds were quantified in the supernatant by UPLC-MS/MS, as described below. CL_int_ was calculated as the product of the compound disappearance rate and incubation volume, normalized by the cell number.

### Compound quantification

Compound quantification was performed using UPLC-MS/MS. The system consisted of a Waters Xevo TQ MS with electrospray ionization coupled to a Waters Acquity UPLC. Compounds were chromatographically separated with a 2 min gradient elution (flow rate of 0.5 ml/min) on a Waters BEH C18 column, 2.1 × 50 mm (1.7 μm) at 60 °C, as described elsewhere^25^. Mass transitions for each compound and their respective cone voltages and collision energies can be found in Supplementary Table 7.

### Protein quantification of drug-transporting proteins and drug-metabolizing enzymes

Protein concentrations for drug-transporting proteins and drug-metabolizing enzymes were quantified as previously described^44^. Briefly, a cell pellet (10 × 10^6^ cells) of freshly isolated human hepatocytes or of cells cultured in monolayer format was lysed with 100 mM Tris/HCl, pH 7.8, 2% (m/v) SDS and 50 mM DTT. Proteins were denatured at 95 °C for 5 min. The lysates were sonicated and then clarified by centrifugation. Trypsin digestion was performed using the filter-aided sample preparation protocol^82^. Peptides were eluted and spiked with stable-isotope labelled standard peptides (JPT Peptide Technologies) and analyzed with UPLC-MS/MS as previously described^44^. The sequences of proteospecific peptides have been previously published^83^,^84^.

### Intracellular compound accumulation simulations

Intracellular compound accumulation was simulated, as previously described^48^, with a mechanistic model developed in R version 3.1.1 (http://www.r-project.org) using the *deSolve* package for differential equation solving. Integration was performed using the *ode* function, which automatically selects an integration algorithm of appropriate stiffness. The model consisted of two compartments (extracellular and intracellular), with model dimensions set to reflect the setup used in accumulation experiments in MDCK cells (apical surface area = 1 × 10^−4^ m^2^, extracellular volume = 200 μl, total intracellular volume = 0.25 μl). In simulations intended to reflect compound accumulation in MDCK P-gp knockout cells, compound transport across the apical membrane was incorporated as a combination of transporter-mediated uptake (assumed to follow regular Michaelis-Menten kinetics) and bidirectional passive Fick’s diffusion. Simulations of compound accumulation in MDCK cells transfected with human P-gp also included a saturable efflux component in the apical membrane, while simulations of compound accumulation in cells where active transport has been inhibited with cyclosporine A included only bidirectional passive Fick’s diffusion. All combinations of low, medium, and high values of passive permeability (0.1, 1, and 10 × 10^−6^ cm/s), and V_max_ (10, 100, and 1000 pmol/min/mg protein) and K_m_ (1, 10, and 100 μM) for each of the transporters were simulated. Each simulation was repeated at 20 concentrations in the range 0.01 μM–100 μM. Drug accumulation was simulated over 120 min, and the resulting equilibrium intra- and extracellular unbound concentrations were used to calculate F_ic_. The concentration dependencies of F_ic_ were then visually inspected to identify scenarios that matched experimental observations.

### Statistics

All experiments were performed in triplicate on at least 2 independent occasions. Atorvastatin and lopinavir were used as controls in intracellular fraction of unbound compound experiments (added to all cassettes: average coefficient of variation (CV) in a single experiment <20%; day-to-day CV < 10%), and in intracellular compound accumulation experiments (in parallel with tested compound: average CV in a single experiment <10%; day-to-day CV < 10%). All results are presented as geometric mean ± standard error of the mean (S.E.M.), unless otherwise stated. A *p*-value < 0.05 was considered significant.

## Additional Information

**How to cite this article:** Mateus, A. *et al*. Intracellular drug bioavailability: a new predictor of system dependent drug disposition. *Sci. Rep.* **7**, 43047; doi: 10.1038/srep43047 (2017).

**Publisher's note:** Springer Nature remains neutral with regard to jurisdictional claims in published maps and institutional affiliations.

## Supplementary Material

Supplementary Information
