# Supplementary material for: Intracellular drug bioavailability: a new predictor of system dependent drug disposition
Source: Sci Rep. 2017 Feb 22;7:43047. doi: 10.1038/srep43047 (PMC5320532; doi:10.1038/srep43047)
Supplement: Supplementary Information [file srep43047-s1.pdf]

Supporting information

Intracellular drug bioavailability: a new predictor of system dependent drug disposition

Mateus, André[1*]; Treyer, Andrea[1*]; Wegler, Christine[1,2]; Karlgren, Maria[1]; Matsson, Pär[1]; Artursson, Per[1,3,4]

[*]These authors contributed equally to this manuscript.

Affiliations:

[1]Department of Pharmacy, Uppsala University, BMC, Box 580, Uppsala SE-751 23, Sweden

[2]Cardiovascular and Metabolic Diseases Innovative Medicines, DMPK, AstraZeneca R&D, Mölndal SE-431 83, Sweden

[3]Uppsala University Drug Optimization and Pharmaceutical Profiling Platform (UDOPP), Department of Pharmacy, Uppsala University, Box 580, Uppsala SE-751 23, Sweden

[4]Science for Life Laboratory Drug Discovery and Development platform (SciLifelab DDD-P), Uppsala University, Uppsala SE-751 23, Sweden

Address correspondence to:

Per Artursson, PhD

Professor in Dosage Form Design

Department of Pharmacy

Uppsala University

Box 580

SE-751 23 Uppsala, Sweden

Email: per.artursson@farmaci.uu.se

Phone: +46 – 18 471 44 71

Fax: +46 – 18 471 44 71

Supplementary results

Using CRISPR-Cas9 to knock-out basal canine P-gp expression in wild-type MDCK cells

To assess the impact of the expression of canine P-gp in wild-type MDCK cells, we compared steady-state cellular uptake (Kp) in these cells and in human P-gp-transfected MDCK cells (Supplementary table 6). We observed that using wild-type MDCK cells as a control might underestimate the impact of P-gp on the translocation of drugs across the cell membrane, as the difference between the two cell lines was similar for substrates and non-substrates of P-gp (Supplementary figure 7). We also considered the use of elacridar (a P-gp inhibitor[85]) in P-gp transfected cells to ablate transport through this protein (both human and canine). This resulted in a difference in uptake of P-gp substrates between P-gp-transfected and cells where transport was inhibited (on average 2.1-fold lower uptake in P-gp transfected cells for substrates (range 0.77- to 27-fold); p <0.001 in Wilcoxon matched-pairs signed rank test; Supplementary figure 8), while no difference was observed for non-substrates (p = 0.10 in Wilcoxon matched-pairs signed rank test). Finally, we considered the use of wild-type MDCK cells where canine P-gp expression was silenced using CRISPR-Cas9 technology[43] as a control cell line. Since we did not observe significant differences between the uptake of compounds in P-gp inhibited cells and in P-gp-CRISPR-Cas9 knock-out cells (p = 0.15 in Wilcoxon matched-pairs signed rank test; Supplementary figure 9), we used the latter cell line throughout this work to establish baseline transport in MDCK cells. In this way the use of chemical inhibition, which can affect other transporters[48], could be avoided. P-gp overexpressing cells used in this study also expressed the canine homolog of this transporter. For the purpose of this study, we considered it unnecessary to silence this transporter as our focus was not to investigate the impact of human P-gp on these substrates, but to study the overall impact of efflux transporters on F_{ic}.

Supplementary figures

*Supplementary figure 1. Influence of physicochemical descriptors (log P (**a**), log D (**b**), PSA (**c**), and MW (**d**)) on differential intracellular bioavailability (F_{ic}) in OATP1B1- and mock-transfected HEK293 cells. Ratio F_{ic} (OATP1B1/mock) corresponds to the ratio between F_{ic} in OATP1B1- and mock-transfected cells. Negatively charged compounds at pH 7.4 are represented as triangles, neutral and zwitterionic species are represented by circles, and positively charged compounds are represented by squares. Substrates of OATP1B1 are highlighted in green.*

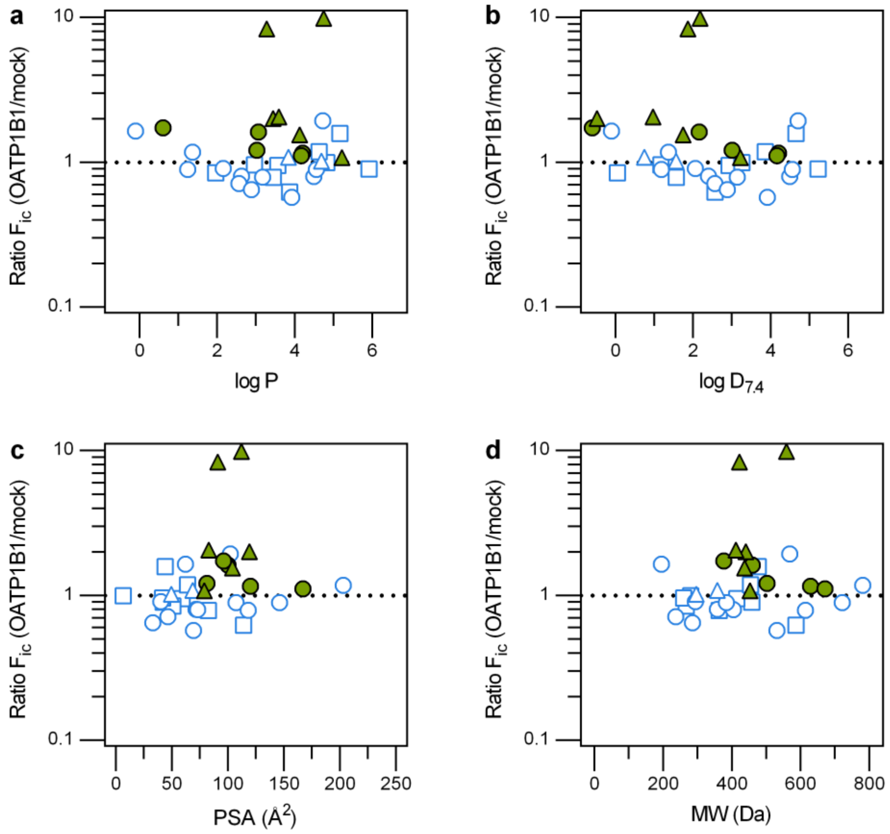

*Supplementary figure 2. Influence of physicochemical descriptors (log P (**a**), log D (**b**), PSA (**c**), and MW (**d**)) on differential intracellular bioavailability (F_{ic}) in P-gp-transfected and P-gp-KO MDCK cells. Ratio F_{ic} (P-gp/KO) corresponds to the ratio between F_{ic} in P-gp-transfected and P-gp-KO cells. Negatively charged compounds at pH 7.4 are represented as triangles, neutral and zwitterionic species are represented by circles, and positively charged compounds are represented by squares. Substrates of P-gp are highlighted in yellow.*

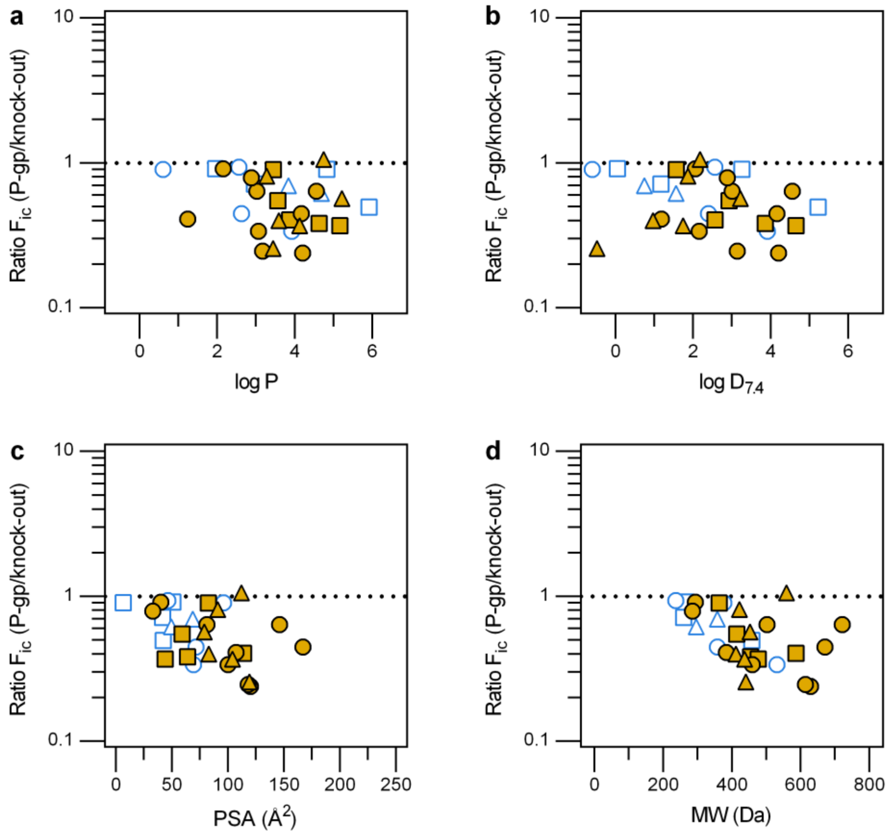

Supplementary figure 3. Relationship between compound concentration and F_{ic} in P-gp-transfected MDCK cells (yellow filled squares), P-gp-KO MDCK cells (blue circles) and P-gp-KO cells incubated with 10 µM cyclosporine A (gray triangles) for loperamide.

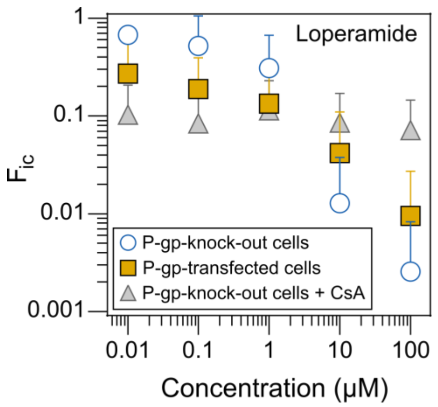

Supplementary figure 4. Relationship between compound concentration and $f_{u,cell}$ in P-gp-KO MDCK cells for simvastatin acid (yellow triangles) and loperamide (blue circles). For simvastatin acid, samples at 0.01 µM and 0.1 µM were below limit of quantification.

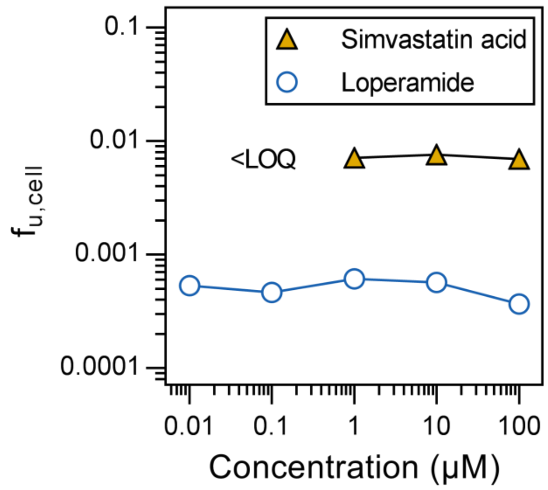

*Supplementary figure 5. Influence of physicochemical descriptors (log P (**a**), log D (**b**), PSA (**c**), and MW (**d**)) on differential intracellular bioavailability (F_{ic}) in monolayer and suspension human hepatocytes. Ratio F_{ic} (monolayer/suspension) corresponds to the ratio between F_{ic} in monolayer and suspension cells. Negatively charged compounds at pH 7.4 are represented as triangles and neutral species are represented by circles.*

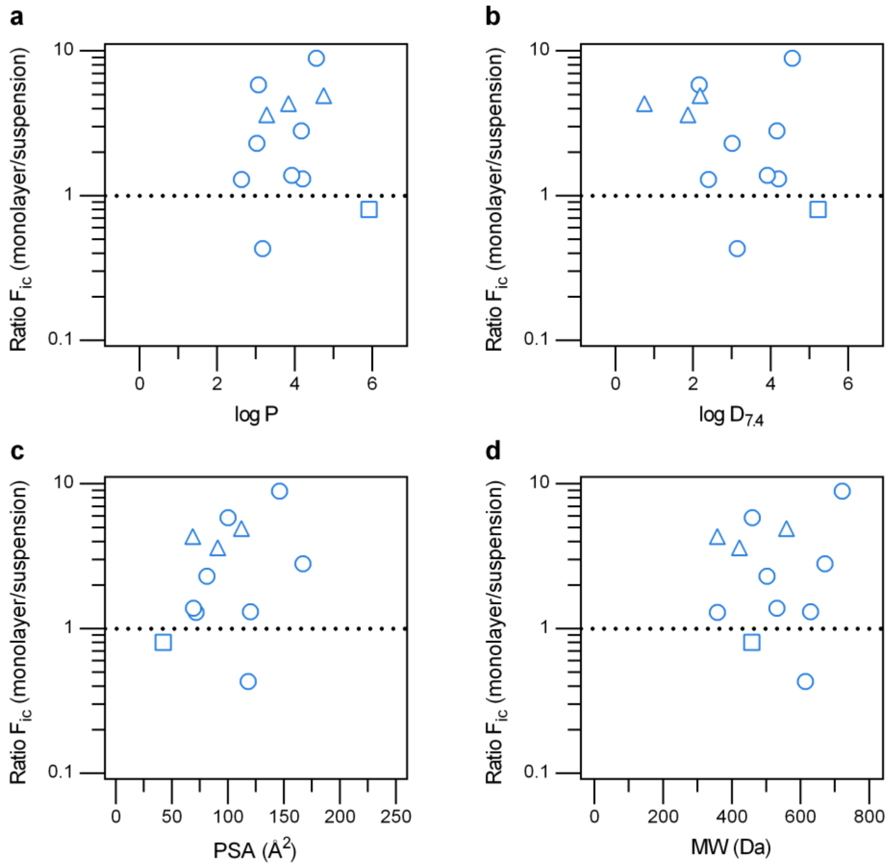

Supplementary figure 6. Transporter and enzyme protein levels measured by mass spectrometry-based targeted proteomics. No significant differences were observed between the two culturing conditions in Wilcoxon matched-pairs signed rank test (p = 0.6).

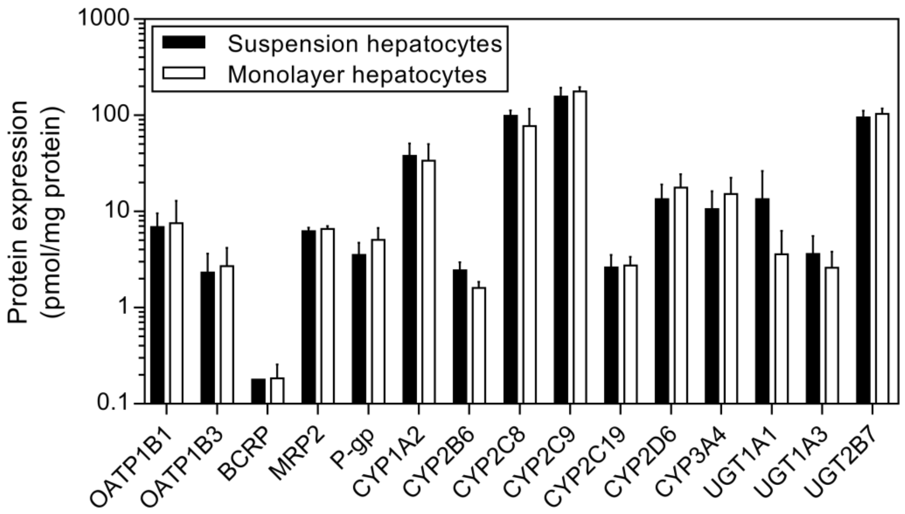

Supplementary figure 7. Influence of basal expression of canine P-gp in wild-type MDCK cells on the steady-state cellular uptake (Kp) of compounds. Negatively charged compounds at pH 7.4 are represented as triangles, neutral and zwitterionic species are represented by circles, and positively charged compounds are represented by squares. Substrates of P-gp are highlighted in yellow.

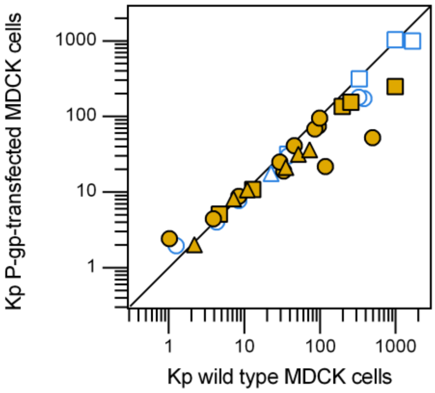

Supplementary figure 8. Influence of P-gp inhibition by elacridar (10 µM) in P-gp transfected MDCK cells on the steady-state cellular uptake (Kp) of compounds. Negatively charged compounds at pH 7.4 are represented as triangles, neutral and zwitterionic species are represented by circles, and positively charged compounds are represented by squares. Substrates of P-gp are highlighted in yellow.

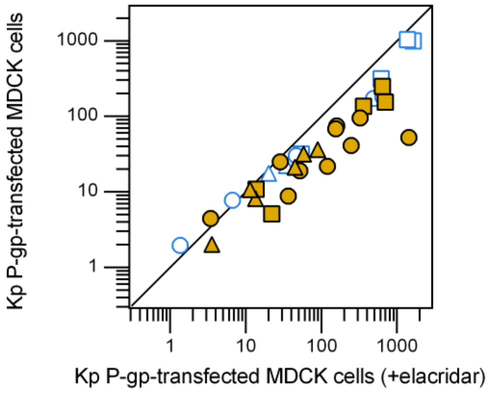

Supplementary figure 9. Comparison of the impact from CRISPR-Cas9 knock-out of canine P-gp and elacridar-mediated chemical inhibition of P-gp on the steady-state cellular uptake (Kp) of compounds. Negatively charged compounds at pH 7.4 are represented as triangles, neutral and zwitterionic species are represented by circles, and positively charged compounds are represented by squares. Substrates of P-gp are highlighted in yellow.

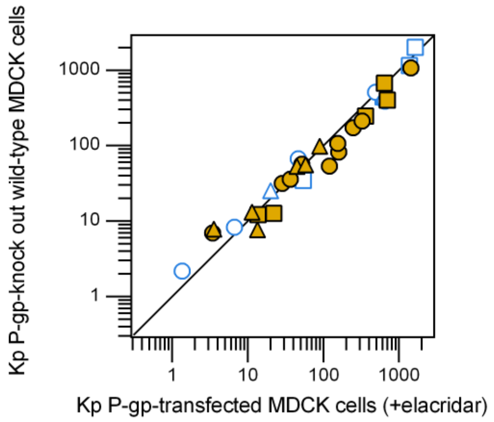

Supplementary tables

Supplementary table 1. Transport and physicochemical characteristics of compound set included in this study.

Compound	OATP1B1 substrate[a]	P-gp substrate[a]	log P[b]	log $D_{7.4}$[b]	PSA[b]	MW[b]
Negatively-charged compounds at pH 7.4						
Atorvastatin	●	●	4.8	2.2	112	559
Candesartan	●	●	3.4	-0.48	119	440
Diclofenac			4.7	1.6	49	296
Fluvastatin	●	●	3.6	1.0	83	411
Indomethacin			3.8	0.74	69	358
Pitavastatin	●	●	3.3	1.9	91	421
Repaglinide	●	●	5.2	3.2	79	453
Simvastatin acid	●	●	4.1	1.7	104	437
Neutral compounds at pH 7.4						
Caffeine			-0.11	-0.11	62	194
Carbamazepine			2.6	2.6	46	236
Diazepam		●	2.9	2.9	33	285
Digoxin		●	1.4	1.4	203	781
Indinavir		●	3.2	3.1	118	614
Ketoconazole			3.9	3.9	69	531
Lopinavir	●	●	4.2	4.2	120	629
Lovastatin			4.5	4.5	73	405
Nelfinavir		●	4.7	4.7	102	568
Ondansetron		●	2.2	2.1	40	293
Prazosin		●	1.2	1.2	107	383
Ritonavir		●	4.6	4.6	146	721
Rosiglitazone			2.6	2.4	72	357
Saquinavir	●	●	4.2	4.2	167	671
Positively-charged compounds at pH 7.4						
Astemizole			5.9	5.2	42	459
Diltiazem		●	3.6	2.9	59	415
Imipramine			4.8	3.3	6	280
Irinotecan		●	3.9	2.6	114	587
Loperamide		●	5.2	4.7	44	477
Metoprolol			2.0	0.05	51	267
Propranolol			3.0	1.2	42	259
Talinolol		●	3.5	1.6	83	364
Verapamil		●	4.6	3.9	64	455
Zwitterionic compounds at pH 7.4						
Cerivastatin	●	●	3.1	2.2	100	460
Enalapril	●		0.60	-0.61	96	376
Fexofenadine	●	●	3.0	3.0	81	502

[a]Collated from literature[74, 75, 76]

[b]Calculated with ADMET Predictor v7.0 (SimulationsPlus)

Supplementary table 2. Cell binding ($f_{u,cell}$), intracellular accumulation (Kp) and intracellular bioavailability (F_{ic}) in HEK293 cells. Results are shown as geometrical mean ± S.E.M.

	mock-transfected HEK293 cells			OATP1B1-transfected HEK293 cells		
**Compound**	$f_{u,cell}$	**Kp**	F_{ic}	$f_{u,cell}$	**Kp**	F_{ic}[a]
Negatively-charged compounds at pH 7.4						
Atorvastatin	0.025±0.004	2.8±0.4	0.072±0.016	0.026±0.002	28±1	0.71±0.12
Candesartan	0.093±0.011	1.4±0.2	0.13±0.02	0.094±0.002	2.8±0.5	0.26±0.06
Diclofenac	0.059±0.007	32±6	1.9±0.4	0.082±0.009	32±7	1.9±0.5
Fluvastatin	0.017±0.003	45±6	0.80±0.17	0.015±0.001	94±12	1.6±0.4
Indomethacin	0.053±0.006	13±2	0.71±0.12	0.065±0.004	14±1	0.77±0.12
Pitavastatin	0.023±0.005	4.2±0.3	0.097±0.024	0.025±0.006	35±3	0.81±0.20
Repaglinide	0.016±0.001	68±8	1.1±0.1	0.019±0.003	73±9	1.1±0.2
Simvastatin acid	0.019±0.003	41±4	0.80±0.14	0.021±0.001	64±6	1.2±0.2
Neutral compounds at pH 7.4						
Caffeine	1.0±0.1	0.73±0.11	0.73±0.12	0.70±0.02	1.2±0.2	1.2±0.2
Carbamazepine	0.22±0.04	7.5±0.9	1.6±0.4	n.d.	5.4±0.6	1.2±0.3
Diazepam	0.033±0.004	34±1	1.1±0.1	n.d.	22±2	0.72±0.11
Digoxin	0.013±0.005	3.4±0.8	0.044±0.019	n.d.	4.0±0.4	0.051±0.019
Indinavir	0.054±0.002	17±1	0.92±0.09	n.d.	13±1	0.73±0.05
Ketoconazole	0.010±0.002	378±85	3.9±1.1	0.012±0.002	217±42	2.2±0.6
Lopinavir	0.0093±0.0027	75±5	0.70±0.21	0.0077±0.0004	87±7	0.81±0.24
Lovastatin	0.0037±0.0008	444±63	1.6±0.4	0.0029±0.0006	355±48	1.3±0.3
Nelfinavir	0.00039±0.00008	337±113	0.13±0.05	0.00044±0.00010	652±228	0.25±0.10
Ondansetron	0.14±0.01	22±3	3.0±0.5	0.11±0.01	20±2	2.7±0.4
Prazosin	0.055±0.002	22±6	1.2±0.3	0.058±0.002	20±3	1.1±0.2
Ritonavir	0.013±0.001	77±10	1.0±0.2	0.016±0.002	68±15	0.90±0.21
Rosiglitazone	0.058±0.004	19±2	1.1±0.1	0.044±0.001	15±2	0.90±0.11
Saquinavir	0.0033±0.0013	272±43	0.89±0.38	0.0027±0.0004	302±96	0.99±0.50
Positively-charged compounds at pH 7.4						
Astemizole	0.0018±0.0006	821±253	1.5±0.6	0.0013±0.0004	738±576	1.3±1.1
Diltiazem	0.055±0.004	43±9	2.4±0.5	0.083±0.005	41±7	2.3±0.4
Imipramine	0.018±0.002	349±96	6.4±1.8	0.016±0.001	345±126	6.3±2.4
Irinotecan	0.028±0.003	8.4±0.8	0.24±0.03	0.042±0.003	5.2±0.4	0.15±0.02
Loperamide	0.0078±0.0008	558±204	4.3±1.7	n.d.	881±215	6.8±1.8
Metoprolol	0.76±0.09	11±1	8.6±1.4	0.22±0.01	9.6±1.1	7.3±1.2
Propranolol	0.032±0.003	131±15	4.2±0.6	0.032±0.002	126±14	4.0±0.6
Talinolol	0.074±0.003	26±4	1.9±0.3	n.d.	20±1	1.5±0.1
Verapamil	0.035±0.002	128±32	4.5±1.1	0.036±0.001	151±17	5.3±0.7
Zwitterionic compounds at pH 7.4						
Cerivastatin	0.018±0.001	34±3	0.62±0.07	0.027±0.001	55±15	1.00±0.28
Enalapril	0.23±0.01	0.50±0.05	0.12±0.01	0.19±0.01	0.86±0.10	0.20±0.02
Fexofenadine	0.049±0.005	1.6±0.2	0.078±0.013	0.094±0.004	1.9±0.2	0.094±0.014

[a]Calculated with $f_{u,cell}$ from mock-transfected HEK293 cells

Supplementary table 3. Cell binding ($f_{u,cell}$), intracellular accumulation (Kp) and intracellular bioavailability (F_{ic}) in MDCK cells. Results are shown as geometrical mean ± S.E.M.

Compound	$f_{u,cell}$ MDCK cells	P-gp-KO (CRISPR) MDCK cells		P-gp-transfected MDCK cells	
		**Kp**	**F_{ic}**	**Kp**	**F_{ic}**
Negatively-charged compounds at pH 7.4					
Atorvastatin	0.027±0.004	7.7±2.1	0.21±0.06	8.1±0.3	0.22±0.03
Candesartan	0.29±0.02	7.8±2.3	2.2±0.7	2.0±0.4	0.57±0.13
Diclofenac	0.033±0.003	36±3	1.2±0.1	22±1	0.74±0.06
Fluvastatin	0.0099±0.0026	53±4	0.53±0.14	21±2	0.21±0.06
Indomethacin	0.025±0.005	25±2	0.63±0.13	18±2	0.44±0.10
Pitavastatin	0.023±0.005	13±1	0.30±0.07	11±2	0.24±0.07
Repaglinide	0.0069±0.0008	56±4	0.39±0.05	32±1	0.22±0.02
Simvastatin acid	0.0097±0.0012	99±3	0.95±0.12	36±2	0.35±0.05
Neutral compounds at pH 7.4					
Caffeine	1.0±0.1	n.d.	n.d.	4.0±1.4	4.0±1.4
Carbamazepine	0.22±0.02	8.4±0.8	1.8±0.2	7.8±0.5	1.7±0.2
Diazepam	0.021±0.004	32±2	0.67±0.13	25±1	0.53±0.10
Digoxin	0.032±0.010	n.d.	n.d.	2.4±0.7	0.079±0.034
Indinavir	0.048±0.004	36±4	1.7±0.2	8.8±0.8	0.42±0.05
Ketoconazole	0.0042±0.0005	511±26	2.2±0.3	173±43	0.73±0.20
Lopinavir	0.0061±0.0013	174±9	1.1±0.2	41±6	0.25±0.06
Lovastatin	0.0020±0.0017	392±23	0.79±0.66	179±48	0.36±0.32
Nelfinavir	0.00010±0.00004	1078±50	0.11±0.04	53±19	0.0054±0.0027
Ondansetron	0.031±0.003	83±6	2.5±0.3	75±9	2.3±0.4
Prazosin	0.025±0.002	54±7	1.3±0.2	22±4	0.54±0.10
Ritonavir	0.0093±0.0033	108±35	1.0±0.5	69±4	0.64±0.23
Rosiglitazone	0.021±0.003	67±11	1.4±0.3	30±4	0.65±0.13
Saquinavir	0.0028±0.0011	214±35	0.61±0.27	96±10	0.27±0.11
Positively-charged compounds at pH 7.4					
Astemizole	0.000010±0.000020	2013±200	0.021±0.040	995±94	0.010±0.020
Diltiazem	0.012±0.002	247±14	3.1±0.6	136±42	1.7±0.6
Imipramine	0.00089±0.00122	1153±62	1.0±1.4	1039±217	0.93±1.28
Irinotecan	0.038±0.003	13±1	0.48±0.05	5.1±0.2	0.19±0.02
Loperamide	0.00051±0.00051	675±161	0.34±0.35	249±43	0.13±0.13
Metoprolol	0.080±0.005	35±3	2.8±0.3	32±5	2.5±0.4
Propranolol	0.0037±0.0017	441±34	1.6±0.7	314±73	1.2±0.6
Talinolol	0.056±0.005	12±1	0.68±0.09	11±2	0.61±0.10
Verapamil	0.0046±0.0016	403±9	1.9±0.6	154±25	0.71±0.27
Zwitterionic compounds at pH 7.4					
Cerivastatin	0.010±0.004	57±10	0.57±0.22	19±1	0.19±0.07
Enalapril	1.0±0.0	2.2±0.4	2.2±0.4	2.0±0.6	2.0±0.6
Fexofenadine	0.056±0.006	7.0±0.2	0.39±0.04	4.4±0.5	0.25±0.04

Supplementary table 4. Cell binding ($f_{u,cell}$), intracellular accumulation (Kp) and intracellular bioavailability (F_{ic}) in freshly isolated human hepatocytes. Results are shown as geometrical mean ± S.E.M.

		Suspension hepatocytes		Monolayer hepatocytes	
**Compound**	**$f_{u,cell}$ hepatocytes**	**Kp**	**F_{ic}**	**Kp**	**F_{ic}**
Negatively-charged compounds at pH 7.4					
Atorvastatin	0.0089±0.0024	94±17	0.83±0.28	346±48	3.1±0.9
Fluvastatin	0.013±0.005	188±62	2.4±1.2	746±116	9.4±4.0
Indomethacin	0.048±0.012	50±17	2.4±1.0	49±4	2.3±0.6
Pitavastatin	0.017±0.011	60±15	1.0±0.7	118±10	2.0±1.3
Simvastatin acid	0.0030±0.0013	94±21	0.28±0.13	1245±146	3.7±1.6
Neutral compounds at pH 7.4					
Indinavir	0.032±0.008	19±5	0.62±0.22	10±1	0.32±0.09
Ketoconazole	0.0061±0.0011	1057±164	6.4±1.6	1463±807	8.9±5.2
Lopinavir	0.0040±0.0018	89±15	0.35±0.17	126±30	0.50±0.26
Ritonavir	0.0039±0.0005	175±54	0.68±0.23	616±151	2.4±0.7
Rosiglitazone	0.020±0.006	152±12	3.1±0.9	196±17	3.9±1.2
Saquinavir	0.0051±0.0047	117±33	0.59±0.57	432±159	2.2±2.2
Positively-charged compounds at pH 7.4					
Astemizole	0.00036±0.00012	1991±1094	0.73±0.47	1598±308	0.58±0.22
Imipramine	0.0036±0.0002	218±51	0.77±0.19	4350±609	15±2
Propranolol	0.013±0.001	66±16	0.88±0.23	1545±87	21±2
Zwitterionic compounds at pH 7.4					
Cerivastatin	0.014±0.003	230±44	3.2±0.9	789±22	11±2
Fexofenadine	0.095±0.016	3.4±0.2	0.32±0.06	9.4±0.3	0.89±0.16

Supplementary table 5. Summary of interactions of subset of compounds against a panel of important drug-transporting proteins and metabolizing enzymes[74, 75, 76]. Black dots represent compounds that are substrates of transporter or enzyme.

Compound	OATP1B1	OATP1B3	BCRP	MRP2	MRP4	P-gp	CYP1A2	CYP2B6	CYP2C8	CYP2C9	CYP2C19	CYP2D6	CYP3A4	UGT1A1	UGT1A3	UGT2B7
Negatively-charged compounds at pH 7.4																
Atorvastatin	●	●		●	●	●			●				●	●	●	●
Fluvastatin	●	●	●			●			●	●	●	●	●	●	●	●
Indomethacin										●	●			●		●
Pitavastatin	●		●	●		●			●	●					●	●
Simvastatin acid	●		●	●		●			●			●	●	●	●	
Neutral compounds at pH 7.4																
Indinavir				●		●							●			
Ketoconazole													●			
Lopinavir	●					●							●			
Ritonavir				●		●	●	●				●	●			
Rosiglitazone									●	●						
Saquinavir	●			●		●						●	●			
Positively-charged compounds at pH 7.4																
Astemizole												●	●			
Imipramine							●	●			●	●	●			
Propranolol							●				●	●	●			
Zwitterionic compounds at pH 7.4																
Cerivastatin	●		●	●		●			●				●			
Fexofenadine	●					●										

Supplementary table 6. Intracellular accumulation (Kp) and intracellular bioavailability (F_{ic}) in MDCK cells. Results are shown as mean ± S.E.M.

	**MDCK II wild-type cells**		**P-gp-transfected MDCK cells (+ elacridar)**	
**Compound**	**Kp**	**F_{ic}**	**Kp**	**F_{ic}**
Negatively-charged compounds at pH 7.4				
Atorvastatin	7.2±0.4	0.20±0.03	13±0	0.37±0.05
Candesartan	2.2±0.3	0.63±0.09	3.5±1.2	1.0±0.3
Diclofenac	28±4	0.93±0.16	34±6	1.1±0.2
Fluvastatin	35±5	0.35±0.10	45±6	0.44±0.13
Indomethacin	22±2	0.56±0.12	20±1	0.50±0.10
Pitavastatin	11±0	0.25±0.06	11±1	0.26±0.06
Repaglinide	52±5	0.36±0.05	58±5	0.40±0.05
Simvastatin acid	72±6	0.70±0.10	89±4	0.86±0.12
Neutral compounds at pH 7.4				
Caffeine	n.d.	n.d.	n.d.	n.d.
Carbamazepine	8.4±0.3	1.9±0.1	6.7±0.3	1.5±0.1
Diazepam	29±2	0.62±0.12	28±1	0.60±0.11
Digoxin	n.d.	n.d.	n.d.	n.d.
Indinavir	8.4±0.8	0.40±0.05	36±1	1.7±0.1
Ketoconazole	377±44	1.6±0.3	486±39	2.1±0.3
Lopinavir	45±8	0.28±0.08	247±30	1.5±0.4
Lovastatin	325±38	0.66±0.55	647±73	1.3±1.1
Nelfinavir	494±74	0.051±0.019	1432±145	0.15±0.05
Ondansetron	95±5	2.9±0.3	159±8	4.9±0.5
Prazosin	118±7	2.9±0.3	120±11	3.0±0.4
Ritonavir	85±3	0.79±0.28	154±7	1.4±0.5
Rosiglitazone	38±7	0.81±0.20	46±1	0.99±0.14
Saquinavir	98±9	0.28±0.12	322±18	0.92±0.37
Positively-charged compounds at pH 7.4				
Astemizole	1678±71	0.018±0.033	1643±59	0.017±0.032
Diltiazem	197±17	2.4±0.5	360±12	4.5±0.8
Imipramine	982±123	0.88±1.20	1373±167	1.2±1.7
Irinotecan	4.7±0.4	0.18±0.02	22±1	0.84±0.07
Loperamide	987±198	0.50±0.51	644±95	0.33±0.33
Metoprolol	37±2	3.0±0.2	54±2	4.3±0.3
Propranolol	330±13	1.2±0.5	615±17	2.3±1.0
Talinolol	13±1	0.72±0.10	14±0	0.77±0.07
Verapamil	257±35	1.2±0.4	697±17	3.2±1.1
Zwitterionic compounds at pH 7.4				
Cerivastatin	33±2	0.33±0.12	52±12	0.52±0.22
Enalapril	1.3±0.2	1.3±0.2	1.4±0.2	1.4±0.2
Fexofenadine	3.9±0.3	0.22±0.03	3.4±0.3	0.19±0.03

Supplementary table 7. Mass spectrometric conditions for quantification of compounds.

Compound	Parent m/z	Cone voltage	Daughter m/z	Collision energy	Ionization mode
Astemizole	459.4	42	135.1	38	ESI+
Atorvastatin	559.2	30	440.2	22	ESI+
Caffeine	195.0	32	138.0	18	ESI+
Candesartan	441.4	18	263.2	12	ESI+
Carbamazepine	237.0	30	189.0	32	ESI+
Cerivastatin	460.3	52	356.3	36	ESI+
Diazepam	285.1	42	154.0	26	ESI+
Diclofenac	294.0	18	250.0	15	ESI-
Digoxin	779.5	54	475.1	38	ESI-
Diltiazem	415.2	20	178.0	26	ESI+
Enalapril	377.4	26	234.2	20	ESI+
Fexofenadine	502.4	40	171.1	36	ESI+
Fluvastatin	410.4	26	348.3	16	ESI-
Imipramine	281.3	22	86.0	16	ESI+
Indinavir	614.3	40	97.0	50	ESI+
Indomethacin	358.1	22	139.0	20	ESI+
Irinotecan	587.5	54	124.1	36	ESI+
Ketoconazole	531.4	50	81.9	42	ESI+
Loperamide	477.3	32	210.2	52	ESI+
Lopinavir	629.5	22	155.1	46	ESI+
Lovastatin	405.4	18	199.2	12	ESI+
Metoprolol	268.2	26	116.0	18	ESI+
Nelfinavir	568.5	40	135.0	54	ESI+
Ondansetron	294.3	34	170.1	26	ESI+
Pitavastatin	422.3	38	290.2	28	ESI+
Prazosin	384.3	42	94.9	44	ESI+
Propranolol	260.2	36	116.0	18	ESI+
Repaglinide	453.3	32	230.2	28	ESI+
Ritonavir	721.5	28	140.1	66	ESI+
Rosiglitazone	358.2	38	135.1	26	ESI+
Saquinavir	671.6	46	128.1	78	ESI+
Simvastatin acid	435.3	34	319.2	16	ESI-
Talinolol	364.3	30	308.3	18	ESI+
Verapamil	455.5	40	165.1	28	ESI+
